# Tyrosinase as a multifunctional reporter gene for Photoacoustic/MRI/PET triple modality molecular imaging

**DOI:** 10.1038/srep01490

**Published:** 2013-03-19

**Authors:** Chunxia Qin, Kai Cheng, Kai Chen, Xiang Hu, Yang Liu, Xiaoli Lan, Yongxue Zhang, Hongguang Liu, Yingding Xu, Lihong Bu, Xinhui Su, Xiaohua Zhu, Shuxian Meng, Zhen Cheng

**Affiliations:** 1 Molecular Imaging Program at Stanford (MIPS), Department of Radiology and Bio-X Program, Stanford University, Stanford, California, USA; 2 Department of Nuclear Medicine, Union Hospital, Tongji Medical College, Huazhong University of Science and Technology, Hubei Province Key Laboratory of Molecular Imaging, Wuhan, China

## Abstract

Development of reporter genes for multimodality molecular imaging is highly important. In contrast to the conventional strategies which have focused on fusing several reporter genes together to serve as multimodal reporters, human tyrosinase (TYR) – the key enzyme in melanin production – was evaluated in this study as a stand-alone reporter gene for *in vitro* and *in vivo* photoacoustic imaging (PAI), magnetic resonance imaging (MRI) and positron emission tomography (PET). Human breast cancer cells MCF-7 transfected with a plasmid that encodes TYR (named as MCF-7-TYR) and non-transfected MCF-7 cells were used as positive and negative controls, respectively. Melanin targeted N-(2-(diethylamino)ethyl)-^18^F-5-fluoropicolinamide was used as a PET reporter probe. *In vivo* PAI/MRI/PET imaging studies showed that MCF-7-TYR tumors achieved significant higher signals and tumor-to-background contrasts than those of MCF-7 tumor. Our study demonstrates that TYR gene can be utilized as a multifunctional reporter gene for PAI/MRI/PET both *in vitro* and *in vivo*.

Multimodality molecular imaging research has been developing and flourishing at an unprecedented pace in recent years. It has been one of the major focuses in the field of molecular imaging and has attracted many researchers from a variety of disciplines. Multimodality molecular imaging is generally considered as the incorporation of two or more imaging modalities with the purpose of taking advantage of the strengths while overcoming the weaknesses of each modality. A variety of modalities have been explored for multimodality imaging, including nuclear modalities such as positron emission tomography (PET) and single photon emission computed tomography (SPECT), magnetic resonance imaging (MRI), optical imaging such as bioluminescence imaging (BLI) and fluorescence imaging and more. Currently, there are three important areas of multimodality molecular imaging research: multimodality instrumentations for image collection, software tools for image construction/registration and multimodality imaging agents for obtaining molecular information of diseases^1^.

Reporter gene/reporter probe is an elegant strategy as well as a powerful tool in molecular imaging, allowing one to identify a specific cell population, track cells *in vivo*, monitor drug treatment efficacy, etc.^2^,^3^,^4^. The most commonly used reporter gene/reporter probe systems in molecular imaging field include the herpes simplex virus type-1 thymidine kinase (HSV-tk1) and the corresponding PET probes 9-(4-^18^F-fluoro-3-[hydroxymethyl]butyl)guanine (^18^F-FHBG) and 2′-^18^F-fluoro-5-ethyl-1-β-D-arabinofuranosyluracil (^18^F-FEAU)^5^, the green fluorescent protein (GFP) and red fluorescent protein (RFP) for fluorescence imaging^6^,^7^ and luciferase/luciferin for BLI^8^. In alignment with the extensive efforts on developing multimodality imaging techniques, research on multimodal reporter genes has been actively pursued over the past few years. Considering each imaging modality possesses its own characteristics and limitations, a carefully designed multimodal reporter gene/reporter probe system should allow one to select an appropriate imaging modality for a particular application, obtain simultaneous anatomic and functional disease information, cross-validate information captured by each imaging modality and facilitate translation of preclinical imaging techniques to the clinic^9^.

With regard to performing multimodal reporter gene imaging, a widely-employed strategy is to fuse two or more reporter genes into one recombinant DNA construct (e.g. HSV-tk1-GFP-Fluc); the expressed fusion protein can then serve as a multimodal reporter and be imaged by multiple imaging modalities^7^,^10^,^11^. Although this strategy has been successfully used for many applications, it does suffer from some significant limitations. To name a few, fusion proteins require paired molecular probes or substrates for each imaging modality; fusion genes generally have a large size and are usually difficult to construct; fusion proteins may also suffer from loss of some bioactivity^9^. Therefore, there have been urgent needs to develop new strategies for construction of reporter genes for multimodal imaging.

Instead of using fusion reporter genes for multimodality imaging, a single reporter gene which can be imaged by several modalities represents a new strategy in multimodal imaging and may overcome some of the aforementioned problems. Tyrosinase (TYR) is the primary enzyme responsible for the production of melanin from tyrosine and this enzyme alone is sufficient to promote the melanin production in non-melanogenic cells^12^,^13^. In this study, we hypothesize that TYR gene can be used as the single gene for multimodality molecular imaging. Our basic strategy is simple – TYR is introduced into cells through gene transfer; then the expressed tyrosinase catalyzes the synthesis of melanin from tyrosine precursors. The resulting gene product, melanin, can then be imaged by three modalities including photoacoustic imaging (PAI), MRI and PET (Fig. 1). Firstly, PAI can be achieved because melanin has a broad optical absorption spectrum and is therefore an excellent agent for photoacoustic effect which in turn can be utilized to perform PAI^12^. Secondly, melanin has the ability to chelate metal ions (e.g. Fe^3+^) which provides contrast for MRI^13^. Lastly, PET can be realized through introducing the melanin avid probes such as N-(2-(diethylamino)ethyl)-^18^F-5-fluoropicolinamide (^18^F-P3BZA, see Fig. 1) as a PET reporter probe^11^,^12^. Overall, our goal in this study is to demonstrate the feasibility of using TYR as a single reporter gene for multimodality imaging, both *in vitro* and *in vivo*.

## Results

### *In vitro* TYR introduction and melanin expression in MCF-7-TYR cells

Plasmid vector pc DNA 3.1-TYR was successfully constructed to express TYR, and stable breast cancer cell line expressing TYR was successfully selected by geneticin and named as MCF-7-TYR. In order to measure expression of the gene product TYR in MCF-7-TYR and MCF-7 cells, Western blot analysis was performed on the cell lysates. It was found that the TYR was successfully expressed in MCF-7-TYR cells (Fig. 2A, left lane) but not in the control MCF-7 cells (Fig. 2A, right lane). The activity of TYR in MCF-7-TYR cells, positive control melanotic melanoma cells (B16F10) and negative control cells (MCF-7) was measured using their cell lysates, which were incubated with 1 mg/mL of L-DOPA solution for different time frames. It was found that the amount of dopachrome produced in MCF-7-TYR and B16F10 cells increased over time, while no dopachrome produced in MCF-7 cells. TYR activity in MCF-7-TYR cells was significantly higher than that of B16F10 and MCF-7 cells (*P* < 0.05 for all time points) (Fig. 2B).

The pellets of MCF-7-TYR, B16F10 and MCF-7 cells were also collected to observe their color and melanin expression. The digital photos demonstrated dark colors for both MCF-7-TYR and B16F10 cells while MCF-7 cells showed a light color (Fig. 2C, upper row). Melanin contents of those cells were also measured through quantification analysis of its absorbance at 405 nm. Consistent with the visual examination, the order of melanin content was calculated to be MCF-7-TYR cells > B16F10 > MCF-7 (Fig. 2D). Moreover, pretreatment of melanotic MCF-7-TYR and B16F10 cells with L-tyrosine (2.0 mM) for 24 h significantly enhanced their color and melanin production presumably secondary to the ability of L-tyrosine to promote the melanin production in melanin producing cells^14^. In contrast, no significant change was found for MCF-7 cells treated with or without L-tyrosine (Fig. 2C bottom row, and Fig. 2D). Lastly, in order to perform a preliminary evaluation of the potential impact of knocking in TYR gene into the cells, the viability and growth of MCF-7-TYR and MCF-7 cells were studied by the MTT assay. No significant differences were observed for the growth rate between these two types of cells (*P* > 0.05, Fig. 2E).

### *In vitro* detection of melanin producing cells by PAI

The photocoustic mode images showed the photoacoustic signals of different concentrations of cells ranged from 0.05 to 10 million/mL. Ultrasound images displayed the shape of cell samples in gel phantoms. The cell samples were located at ~3 mm underneath the surface of gel phantoms. As shown in Fig. 3A, photoacoustic signals could be easily detected in both MCF-7-TYR and B16F10 cells. The signal decreased with the reduced concentration of melanotic cells; as low as 2.5 × 10^3^ MCF-7-TYR cells and 2.5 × 10^4^ B16F10 cells could be clearly imaged, while negative control cells MCF-7 could not produce detectable photoacoustic signal even at 10 million/mL, which is expected. Results of quantitative analysis of photoacoustic signal from MCF-7-TYR and B16F10 cells are shown in Fig. 4A. It provides a verification that photoacoustic signal increases along with the higher concentrations of melanotic cells.

### *In vitro* detection of melanin producing cells by MRI

Three different cell concentrations were used to study the sensitivity of MRI for detection of melanotic cell lines (MCF-7-TYR, B16F10) and the amelanotic cell line (MCF-7). It was found that MCF-7-TYR and B16F10 cells cultured with FeCl_3_-enriched medium displayed much higher signals on T1-weighted MR images, compared with MCF-7 cells (Fig. 3B). High signal could be clearly seen at a concentration of 2.5 × 10^7^/mL (2.5 million cells) for both MCF-7-TYR and B16F10 cells. Moreover, it was also observed that cells cultured in medium without FeCl_3_-enrichment could not produce detectable T1-weighted signal for all three cell lines (Fig. 3B). Quantification results shown in Fig. 4B revealed that the MR signal of MCF-7-TYR and B16F10 increased slightly along with the increased concentration of cells without FeCl_3 _treatment; the signal improved dramatically, however, in the melanotic cells pretreated with FeCl_3_. As a comparison, the signal of MCF-7 cells remained consistent in all concentrations without FeCl_3 _pretreatment; with FeCl_3_ treatment the signal increased slightly.

### Cell uptake of ^18^F-P3BZA

Uptake levels of ^18^F-P3BZA in MCF-7-TYR, B16F10 and MCF-7 cells at 0.5 and 1 h are shown in Fig. 3C. ^18^F-P3BZA quickly accumulated in both melanotic cell lines, MCF-7-TYR and B16F10, and reached 5.57 ± 0.31% and 6.85 ± 0.08% at 0.5 h incubation time, respectively. The uptake value continued to increase at 1 h, with a value of 7.14 ± 0.18% for MCF-7-TYR and 8.55 ± 0.24% for B16F10. In comparison, for control MCF-7 cells no significant accumulation of ^18^F-P3BZA was observed and the uptake values were only 0.25 ± 0.05% and 0.32 ± 0.07% at 0.5 and 1 h, respectively.

### *In vivo* multimodality imaging studies

The digital photos of mice bearing melanotic MCF-7-TYR and amelanotic MCF-7 tumors are shown in Fig. 5A and the mice (n = 5 for each group) were subsequently imaged by PAI, MRI and PET.

#### PAI

*In vivo* PAI results are shown in Fig. 5B. An intensive photoacoustic signal could be clearly observed in MCF-7-TYR tumors (upper row) while much lower signal was seen in MCF-7 tumors. The PAI signals at the edge of the MCF-7 tumor mainly resulted from the tumor vasculature. Ultrasound images demonstrated the morphology of the tumor and it was found that MCF-7-TYR tumors had a higher density compared with MCF-7 tumors (middle row). Further quantification analysis indicated that the PAI signals of MCF-7-TYR and MCF-7 tumors were 83.44 ± 13.81 and 32.98 ± 9.51, respectively (*P* < 0.05) (Fig. 6A).

#### MRI

To demonstrate the use of the TYR and its gene product melanin as a reporter for MRI of tumors, T1-weighted images were obtained from each group of mice bearing MCF-7-TYR and MCF-7 tumors (n = 5). MCF-7-TYR tumors displayed significantly higher signals compared with MCF-7 tumors (Fig. 5C). Quantitative analysis of tumor and muscle signals in MRI images was then performed and the tumor/muscle ratios for two groups of mice were obtained (Fig. 6B). The tumor/muscle ratios for MCF-7-TYR and MCF-7 tumors were 1.43 ± 0.08 and 1.21 ± 0.07, respectively (*P* < 0.05).

#### PET

Decay-corrected coronal (top row) and transaxial (bottom row) small-animal PET images of MCF-7-TYR and MCF-7 tumor–bearing mice at 0.5, 1, and 2 h after tail vein injection of ^18^F-P3BZA are shown in Fig. 5D. MCF-7-TYR tumors were clearly visualized with good tumor-to-background contrast at all time points whereas for MCF-7 tumor uptake was hardly visible. Liver and kidney uptakes were also observed in all animals, which was consistent with our previous finding^15^.

### Biodistribution

The MCF-7-TYR and MCF-7 tumor bearing mice (n = 4 for each group) were sacrificed at 2 h after intravenous injection of ^18^F-P3BZA. Very high uptake of the PET probe (11.80 ± 1.60%ID/g) was found in MCF-7-TYR tumors while low uptakes were found in most normal organs. MCF-7-TYR tumor-to-blood and tumor-to-muscle ratios were 76.28 ± 12.02 and 34.41 ± 6.33, respectively. In comparison, the tumor uptake in MCF-7 model was only 0.52 ± 0.06%ID/g and the tumor-to-blood and tumor-to-muscle ratios were 5.62 ± 1.25 and 2.29 ± 0.64, respectively (Table 1).

## Discussion

In this study, we have successfully demonstrated TYR can be used as a single reporter gene to perform multimodality imaging both *in vitro* and *in vivo*. To our knowledge, this is the first time a single reporter is used to perform PAI, MRI and PET imaging. The rationale of using melanin for multimodality imaging lies in the following considerations.

First of all, PAI is an emerging hybrid molecular imaging modality capable of producing *in vivo* 3D images with higher spatial resolution (up to 500 μm at tissue penetration depth of about 5 cm) than existing optical imaging modalities^16^,^17^,^18^. The physical mechanism behind this non-invasive imaging modality is the photoacoustic effect (i.e. transformation from light to ultrasound). PAI can also provide functional and molecular information of diseases in real time using endogenous or exogenous photoacoustic contrast agents^19^. It is thus highly desirable to develop new imaging probes for PAI. Melanin has a broad optical absorption spectrum with significant absorption at near-infrared (NIR) wavelengths, which allows for good tissue penetration^20^. Because of this unique property melanin has been demonstrated to be an excellent endogenous contrast agent for PAI^12^,^16^,^21^,^22^. Very recently, the use of TYR as a reporter gene for PAI was explored given that TYR is the modulator of the synthesis of melanin^23^,^24^,^25^. However, in the prior studies researchers only used TYR as a single modality reporter (PAI) and the potential of melanin as a multimodality reporter was not investigated. Considering the depth limitation (up to 5 cm) of PAI, adding the imaging capacity of melanin for PET and MRI could render the TYR reporter gene strategy suitable for deep tissue as well. Moreover, in our study, we also systematically studied the sensitivity of PAI and carried out *in vivo* imaging in tumor mice models. High sensitivity and cell quantification capability using PAI was achieved in this study; specifically, the sensitivity was high enough to detect a concentration of MCF-7-TYR cells (5 × 10^4^ cells per milliliter, 2.5 × 10^3^ cells total, Fig. 3A), which suggests that TYR reporter strategy can be used to detect a very small number of cells using PAI.

Secondly, thanks to the many channels within melanin granules melanin has a high affinity for a number of metal ions including Fe^3+^, Mg^2+^ and Ca^2+^ and can thus serve as a transporter of metal ions. In fact, pigmented tissues generally contain a large amount of metal ions *in vivo*^26^,^27^,^28^. Clinical observations have shown that melanotic melanomas are associated with hyperintensity on T1-weighted MRI images^29^,^30^. Therefore, TYR was previously proposed to be a reporter gene for MRI because of the good binding capability of melanin with Fe^3+^. The bound metal ions create a substantial increase in T1 signals and can thus be exploited as a contrast mechanism in MRI^13^,^24^,^31^. However, prior studies of using melanin as a MRI contrast agent were only carried out in cells and therefore an *in vivo* proof-of-concept study becomes important. In our work, we successfully demonstrated that TYR can be used as a MRI reporter both *in vitro* and *in vivo* (Fig. 3B and Fig. 5C) while achieving excellent tumor contrast.

Last but not least, nuclear imaging modalities such as SPECT and PET have very high sensitivity and no limitation of tissue penetration. Recently, benzamide analogs have been shown to be the most promising melanin targeted imaging agents^14^,^15^,^32^. Particularly, ^18^F-P3BZA appears to be a favorable agent because of its easy one-step radiochemical synthesis and excellent *in vivo* performance^15^. Therefore, this probe can be effectively used to detect TYR reporter gene expression through melanin production, which is also supported by results of the cell uptake experiments (Fig. 3C) as well as *in vivo* small animal imaging studies in this study (Fig. 5D).

Traditionally, multimodality reporter genes consist of several reporters fused in one recombinant vector. Combination of optical imaging and PET is the most commonly chosen route for multimodality molecular imaging studies. Some of the examples include triple fusion reporter vector hrl-mrfp-ttk^10^, HSV1-TK/GFP/Fluc^11^, et al. In contrast to the conventional strategy, a single TYR reporter gene was employed for multimodality imaging in this study and many advantages of this new strategy were observed. This reporter gene system possesses a simple structure and can be easily constructed. Of note, a tet-on/tet-off system can also be inserted into TYR reporter gene system to further realize the versatile control of gene expression. Moreover, the expression efficiency of this reporter gene is very high – each TYR is able to produce many melanin molecules. Given that melanin is a polymer and contains various functional groups, this molecule provides multiple nonequivalent binding sites for paramagnetic iron ions^33^,^34^ and is able to bind with a large amount of radioactive probes at the same time, which make MRI and PET feasible. There is also no need to administrate any substrate for *in vivo* PAI and MRI. Additionally, TYR is an endogenous gene and melanin is present in many human pigmented cells and tissues including skin, hair, eye, stria vascularis of the inner ear and brain nuclei^35^. This strategy thus enjoys both high biocompatibility and low measurable impact to the cells, as demonstrated in our cell proliferation assay study (Fig. 2E), which could prove to be very important in potential clinical translations in the near future. Furthermore, Melanin is a pigment and the expression of TYR reporter can be visually assessed through the color of cell pellets. Therefore, colormetric assay can also be used for measurement of TYR reporter expression. This characteristic adds to the simplicity and ease-of-use of the TYR reporter strategy. Besides serving as a reporter gene TYR can also be used as a potential therapeutic gene. Benzamide compounds and melanin-binding peptides labeled with therapeutic radionuclides have been actively pursued for melanin targeted melanoma treatment in recent years^36^,^37^,^38^. The melanin induced by TYR gene in cancer cells can thus be viewed as an attractive target for some of these promising agents. Currently, our group has been actively evaluating whether MCF-7-TYR breast cancer can be effectively treated by radioiodinated benzamides. Last but not least, the TYR melanin reporter system is the first reporter system for PAI/MRI/PET. With the triple modality this system can simultaneously provide molecular information in highly sensitivity (PAI and PET) as well as anatomic information in high resolution (MRI). Of note, clinical and small animal hybrid PET/MRI scanners have recently become commercially available and this TYR reporter system can be easily employed by these new imaging instrumentations.

**Figure 1:**
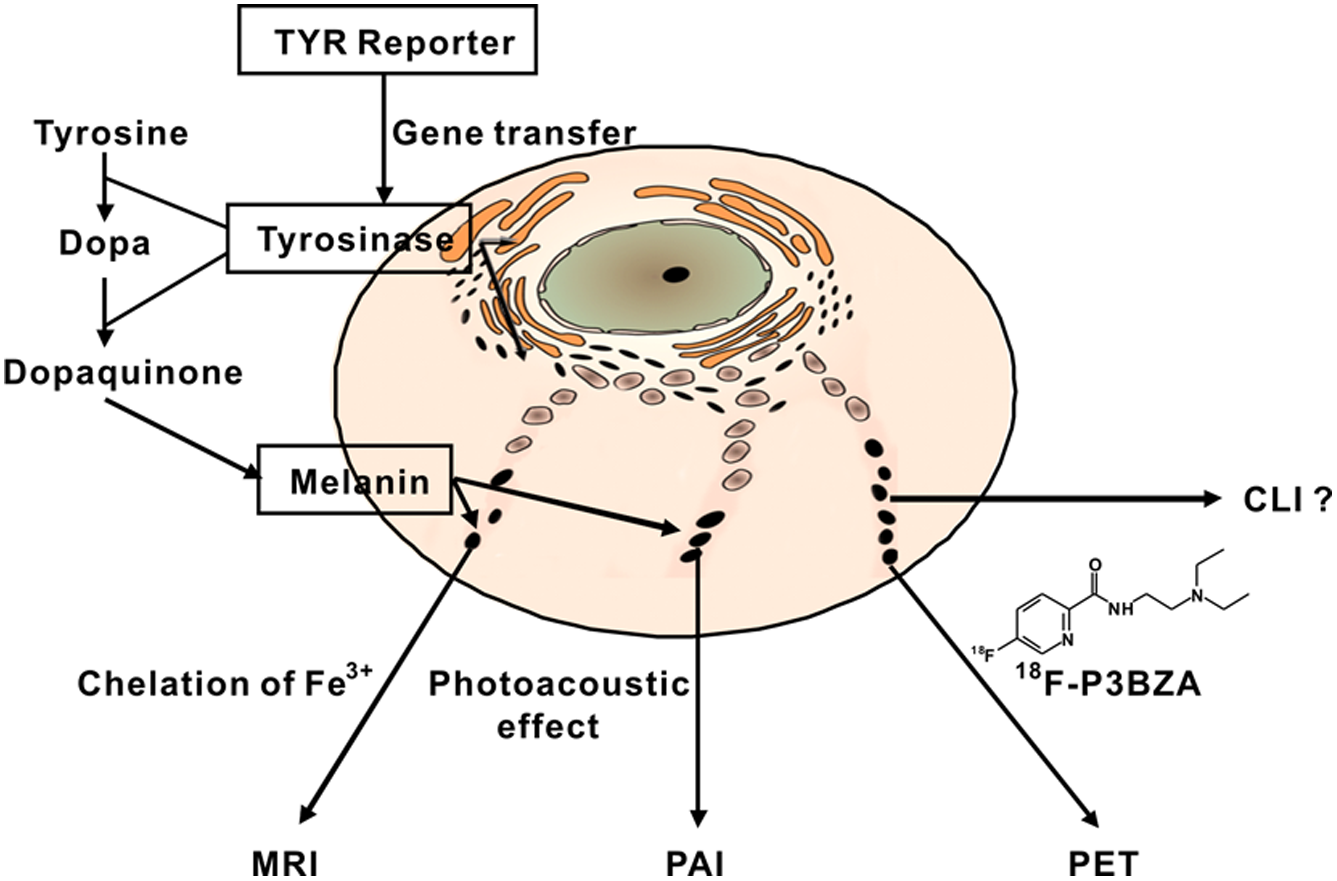
Multimodality molecular imaging of the TYR reporter gene system. TYR reporter is introduced into cells through gene transfer and the expressed tyrosinase catalyzes the oxidation of tyrosine and Dopa to synthesize melanin. Melanin then serves as a multi-functional target. First, melanin has a broad optical absorption and is an excellent agent for photoacoustic effect, which can be used to perform PAI. Second, melanin has the ability to chelate metal ions (Fe^3+^) which provides cantrast for MRI. Third, melanin can be specifically imaged by a recently established melanin avid PET probe, ^18^F-P3BZA. Lastly, PET probe such as ^18^F-P3BZA has the potential to allow Cerenkov Luminesecnce Imaging (CLI) and this application was explored in this work as well.

**Figure 2:**
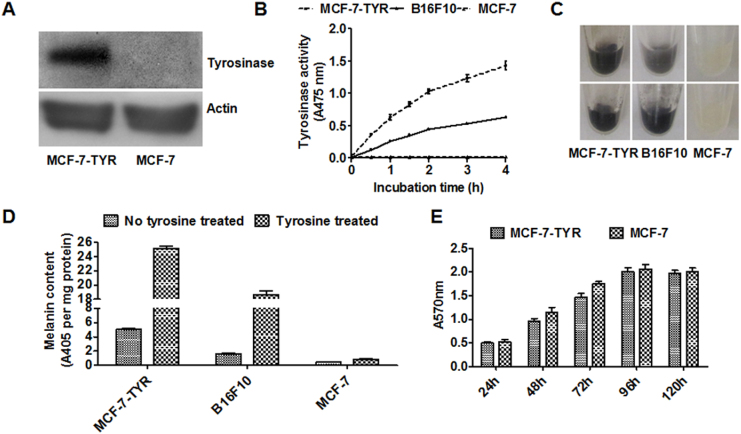
*In vitro* evaluation of the expression of TYR reporter. (A) Western blot assay of tyrosinase expression in MCF-7-TYR and MCF-7 cells. β-actin was used as the control. (B) Time-response tyrosinase activity curves in MCF-7-TYR, B16F10 and MCF-7 cells. (C) Photos of the cell pellets from MCF-7-TYR, B16F10 and MCF-7 cells without (upper) or with (bottom) 2 mM L-tyrosine treatment for 24 h. (D) Melanin production in MCF-7-TYR, B16F10 and MCF-7 cells with or without tyrosine treatment for 24 h. (E) The effect of TYR expression on the growth of the melanotic (MCF-7-TYR) and amelanotic (MCF-7) cells.

**Figure 3:**
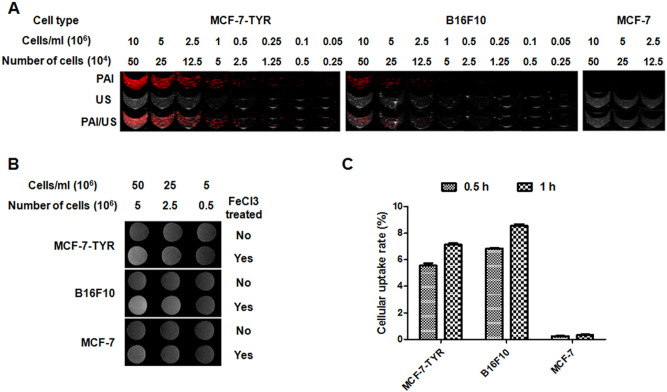
*In vitro* detection of melanotic (MCF-7-TYR, B16F10) and amelanotic (MCF-7) cells. (A) Photoacoustic imaging (top), ultrasound (middle), and PAI/US images (bottom) of the gel phantom with different concentrations of cells. Photoacoustic images were obtained using the Vevo LAZR Photoacoustic Imaging System at a wavelength of 680 nm. (B) MRI images of three concentrations of MCF-7-TYR, B16F10 and MCF-7 cells pretreated without (top row) or with (bottom row) FeCl_3_. (C) Uptake of ^18^F-P3BZA in MCF-7-TYR, B16F10 and MCF-7 cells after incubation with ^18^F-P3BZA at 37°C for 0.5 and 1 h. All results, expressed as percentage of cellular uptake, are mean of triplicate measurements ± SD.

**Figure 4:**
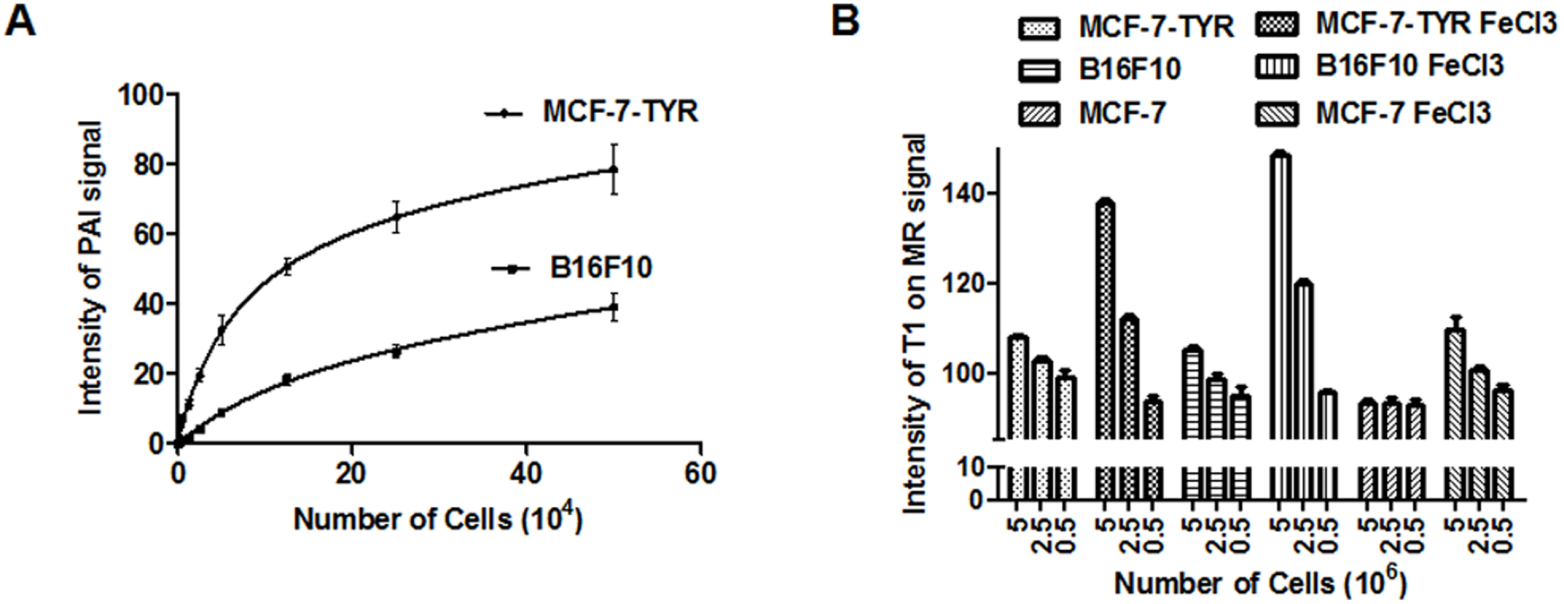
Quantitative analysis of *in vitro* cell phantom PAI and MR imaging results. (A) Photoacoustic signal increased with the higher concentration of melanotic cells. (B) MRI signal of MCF-7-TYR and B16F10 slightly increased with the increased concentrations of cells; a dramatic increase was observed in the melanotic cells pretreated with FeCl_3_. The signal of MCF-7 cells remained consistent in all concentrations without FeCl_3_ pretreatment; with FeCl_3_ treatment the signal increased slightly.

**Figure 5:**
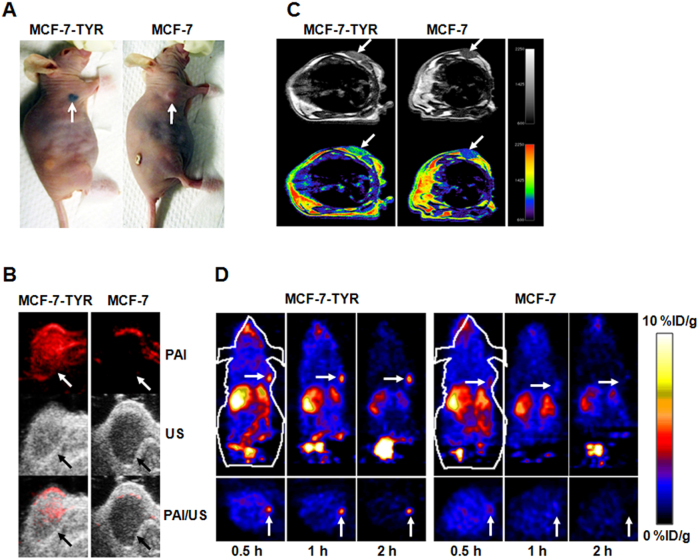
*In vivo* multimodality imaging of tumor bearing mice with PAI, MRI and PET. Five tumor bearing mice were used and tumors were indicated by arrows. (A) Photographic images of tumor bearing mice (left: melanotic MCF-7-TYR tumor; right: amelanotic MCF-7 tumor). (B) PAI (top), ultrasound (middle), and PAI/US images (bottom) of the tumor mice (left: MCF-7-TYR; right: MCF-7). (C) MRI images of MCF-7-TYR (left) and MCF-7 (right) tumors. Top row shows black and white images, and bottom row shows the pseudo-colored images. (D) Representative decay-corrected coronal (top) and transaxial (bottom) small animal PET images of MCF-7-TYR (left three images) and MCF-7 (right three images) tumors acquired at 0.5, 1 and 2 h after tail vein injection of ^18^F-P3BZA. In all three imaging modalities, MCF-7-TYR tumor shows higher contrast than that of MCF-7 tumor.

**Figure 6:**
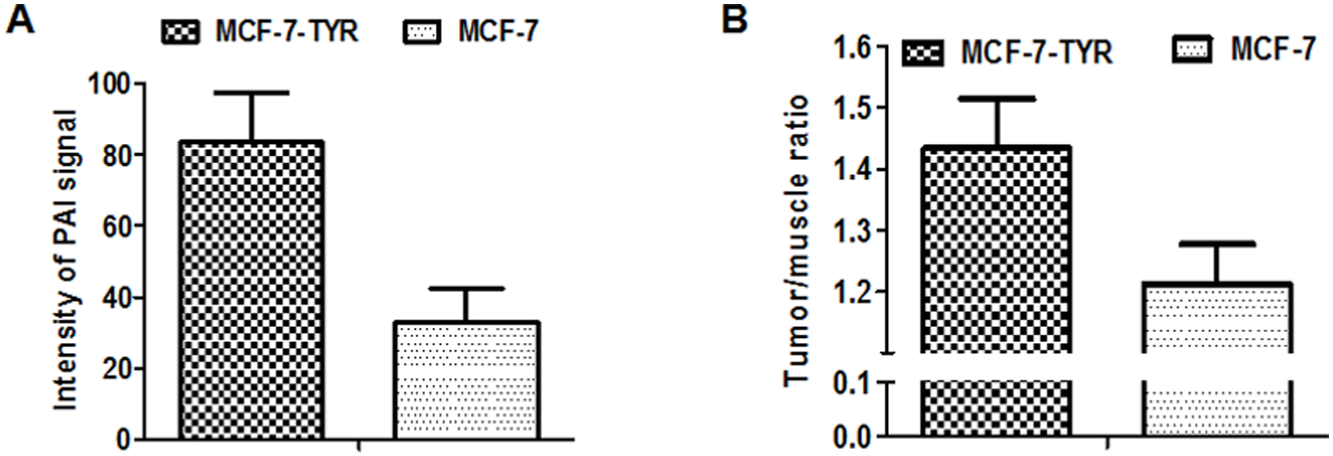
Quantitative analysis of MCF-7-TYR and MCF-7 tumors images obtained from PAI (A) and MRI (B). MCF-7-TYR tumor displays much higher PAI signal than that of MCF-7 tumor, and MRI also demonstrates that higher tumor/muscle ration in MCF-7-TYR than that in MCF-7 model (*P* < 0.05).

Interestingly, researchers have recently found that a variety of radionuclides including ^18^F, ^131^I, ^90^Y, and their labeled compounds can be detected by optical imaging techniques. This finding is mainly attributed to the ability of radioactive materials to produce low energy visible photons, a phenomenon that is named Cerenkov radiation^39^,^40^. This new imaging technique in the molecular imaging was thus appropriately named Cerenkov Luminescence Imaging (CLI) and has quickly found many applications^41^,^42^,^43^,^44^,^45^. In this study, we also explored whether TYR/^18^F-P3BZA reporter gene and reporter probe system can be monitored by CLI (Fig. 1). However, our results showed that although MCF-7-TYR tumors possessed a very high accumulation of ^18^F-P3BZA, the tumors was not detectable by CLI (data not shown). One of the possible explanations could be that melanin has a broad optical absorption spectrum, which absorbs the majority, if not all, of the Cerenkov photons.

In conclusion, this study successfully demonstrates the feasibility of using a single reporter TYR for multimodality molecular imaging. This system enjoys high sensitivity for both PAI and PET; the component of MRI compensates for low spatial resolution of PET and also features a good contrast on T1-weighted images. Therefore, TYR is a highly promising reporter gene system for multimodality molecular imaging in biomedical research. Our study highlights the high potential of using single reporter gene for multimodality imaging.

## Methods

### Construction of TYR expression vector

cDNA encoding human TYR (NM_000372.3) in the pCMV6-XL6 vector was purchased from OriGene Technologies, Inc. (Rockville, MD, USA). TYR DNA was amplified by polymerase chain reaction (PCR) with primers flanking the TYR open reading frame with BamHI and NheI restriction enzyme sequences within the 5′ and 3′ primers, respectively, and it was purified using a Qiagen gel extraction kit (Qiagen, Valencia, CA, USA). The purified TYR encoding the inserted cDNA and the pc DNA 3.1(+) vector (5.4 Kbp; Invitrogen, Carlsbad, CA, USA) were both digested with BamHI and NheI restriction enzymes (New England Biolabs, Inc., Ipswich, MA, USA) and ligated together with T4 DNA ligase (New England Biolabs, Inc., Ipswich, MA, USA). The ligation mixture was used to transform E. Coli DH5α competent cells (Invitrogen, Carlsbad, CA, USA) which were plated on LB broth plates supplemented with 100 μg/mL ampicillin. Bacterial colonies were isolated and plasmid DNA of approximately 7.0 Kbp was isolated from the resulting colonies. After the recombinant plasmid was identified by DNA sequencing and double restriction enzyme digestion, plasmid maxiprep preparation was performed and the concentration of the plasmid was measured. The recombinant expression vector was named as pc DNA 3.1-TYR.

### Generation of MCF-7 cells stably expressing TYR

MCF-7 human breast cancer cell line was originally obtained from American Type Culture Collection (ATCC; Manassas, VA, USA) and cultured in Dulbecco's modified Eagle high-glucose medium (DMEM, Gibco Life Sciences) supplemented with 10% fetal bovine serum (FBS) and maintained in a 37°C, 5% CO_2_ humidified incubator. To establish the MCF-7 cell line producing melanin, MCF-7 cells were transduced with plasmid pc DNA 3.1-TYR using Lipofectamine 2000 (Invitrogen, Carlsbad, CA, USA). One day after transfection, cells were trypsinized and transferred to 10 cm dishes at a ratio of 1:20. In the next day the cells were cultured in DMEM medium with 10% FBS containing 400 μg/mL geneticin. The medium was changed every two or three days. Cells in dishes were grown for several weeks until large cell colonies were visible. Each colony was carefully observed under a light microscope. The colony with the darkest color was considered to be capable of producing melanin and termed as MCF-7-TYR cells. This colony was trypsinized from the dishes, cultured and used for subsequent experiments. B16F10 and non-transfected MCF-7 cells were used as positive and negative control cells, respectively.

### Western blot analysis of tyrosinase protein expression

MCF-7-TYR and MCF-7 cells were washed with phosphate buffered saline (PBS, 0.1 M, pH = 7.4) and lysed in T-PER Tissue Protein Extraction Reagent (Thermo Fisher Scientific Inc., Rockford, IL, USA). Protein contents were determined and equal amount of each protein extract (100 μg per lane) were separated on a 4 – 12% sodium dodecyl sulfate-polyacrylamide gradient gel (SDS-PAGE) by electrophoresis and transferred to a PVDF membrane (Invitrogen, Carlsbad, CA, USA). The membranes were then blocked in 0.1% Tween-20 trisbuffered saline (Tween-TBS) containing 5% non-fat milk for 60 min at room temperature with agitation. The membranes were then incubated overnight at 4°C with the primary antibody diluted in Tween-TBS (tyrosinase monoclonal antibody, 1:200; β-actin polyclonal antibody, 1:5000, respectively, Abcam, Cambridge, MA, USA), and they were washed three times for 10 min each with Tween-TBS, followed by 1 h incubation with horseradish peroxidase-conjugated anti-goat IgG antibody diluted to 1:5000 in Tween-TBS at room temperature. After this, each membrane was washed again. The antigen-antibody peroxidase complex was then detected using enhanced chemiluminescence (ECL) western blotting substrate (Thermo Fisher Scientific Inc., Rockford, IL, USA) according to the manufacturer's instructions, and bands were visualized by exposure to Hyperfilm (Amersham Biosciences, Buckinghamshire, UK).

### Assay of cellular tyrosinase activity

The sample preparation procedure was the same as that described in the Western blot assay. After quantifying the protein levels, the concentration of samples was adjusted to 0.4 μg/μL. A published method with slight modification were then used to measure the tyrosinase activity^46^. Briefly, the experiment was conducted in a 96-well flat-bottom plate. Each well contained 50 μL of cell lysate and 50 μL of 2 mg/mL L-DOPA dissolved in PBS. The final volume of each well was 100 μL, containing 20 μg of protein and 1 mg/mL L-DOPA. The mixture was incubated at 37°C. The absorbance of the reaction mixtures, which correlates with the amount of dopachrome produced, was measured using a plate reader (TECAN, Research Triangle Park, NC, USA) at 475 nm at different time points.

### Measurement of melanin content in cultured cells

Melanin content of MCF-7-TYR, MCF-7, and B16F10 cells was measured as described previously with slight modifications^47^. The cultured cells were pretreated with or without 2 mM L-tyrosine for 24 h, then harvested and washed with PBS. They were sonicated and incubated overnight in 500 μL of 1 N NaOH at room temperature, and the solution was pipetted repeatedly to homogenize the extracts. After determination of protein content, the protein concentration was adjusted to 0.4 μg/μL, and the extracts were then transferred into 96-well plates in duplicate (50 μL aliquots). The relative melanin content of samples was determined by measuring their absorbance at 405 nm. Results were expressed as absorbance of 405 nm per mg protein (A 405 nm/mg protein).

### Cell proliferation assay

Proliferation rate of MCF-7-TYR and MCF-7 cells were evaluated by the MTT assay as described previously^48^. Briefly, cells were seeded in 96-well plates (5,000 cells per well). Following 24, 48, 72, 96 or 120 h incubation, MTT (20 μL, 5 mg/mL in PBS; Promega, Madison, WI, USA) was added, and the plates were incubated at 37°C in 5% CO_2_ for 4 h. Subsequently, medium was discarded and dimethyl sulfoxide (DMSO) was added. The plates were then incubated for 10 min with continuous shaking, and the absorbance of sample was measured at 570 nm using the plate reader.

### *In vitro* MRI of cell phantoms

The agarose based phantoms were prepared using the 300 μL PCR tubes. The bottom of the tubes were filled with 1% UltraPure™ agarose gel (Invitrogen, Carlsbad, CA, USA) in bi-distilled water. After being cooled down, different concentrations of cells (100 μL, ranging from 50 million/mL to 5 million/mL) suspended in 1% agarose were filled into the middle part of the tubes, and then the tops of the tubes were filled with 1% agarose. MRI was performed at the Small Animal Imaging Facility at Stanford University using an Agilent Discovery MR901 System (Agilent Technologies, Inc., Santa Clara, CA, USA). A 72 mm Agilent radiofrequency (RF) coil was used. The imaging protocol consisted of localizer and axial T1-weighted fast spin echo (FSE) sequence with the following parameters: repetition time (TR): 750 ms; echo time (TE): min full; field of view (FOV): 3.0 × 3.0; matrix size: 256 × 256; slice thickness: 1 mm. Image analysis was performed using ImageJ. The contrast was adjusted and regions of interest (ROIs) were drawn over the samples, and the signal of ROIs was then measured.

### *In vitro* PAI and image analysis

The cell phantoms were prepared as following: the 96-well PCR plates were embedded in a cuboid container filled with 1% agarose gel. After solidification, the tubes were pulled out to generate holes, and then the bottoms of the holes were filled with 100 μL of 1% agarose. Different concentrations of cells (50 μL) ranging from 10 million/mL to 0.05 million/mL suspended in 1% agarose were filled into the middle part of the holes. The surface of the phantom was covered with thin 1% agarose gel to make it smooth. The Vevo LAZR PAI System (VisualSonics Inc., Toronto, Canada) with a laser at excitation wavelength of 680 nm and a focal depth of 10 mm was used to acquire photoacoustic and ultrasound images. Image analysis was carried out using ImageJ. Briefly, quantification analysis was performed on the PAI images. All slices of a sample were stacked by Z-Project with the maximum intensity, and ROIs were drawn over the cell sample on the stacked PAI images. The PAI signal intensity was then measured using the ROIs manager tool.

### *In vitro* cell uptake studies

Preparation of ^18^F-P3BZA was conducted the same as described in our previous publication^15^. The cellular uptake studies were performed on MCF-7-TYR, MCF-7 and B16F10 cells using the slightly modified procedure as reported previously^49^,^50^. Briefly, cells (0.2 × 10^6^ per well) were seeded in 12-well plates and incubated overnight at 37°C. Cells were then incubated with 0.5 mL of DMEM medium containing 3.7 kBq (0.1 μCi) of ^18^F-P3BZA at 37°C. At 0.5 or 1 h post-incubaion, the medium was removed and cells were washed 3 times with PBS (pH 7.4) and lysed with 0.1% SDS-0.02 M NaOH for 5 min at room temperature. The radioactivity of the cell lysate was measured by a gamma counter. The percentage of uptake was calculated according to the radioactive counts of cells divided by the total radioactive counts added.

### Subcutaneous tumor models

All animal studies were carried out in compliance with federal and local institutional rules and approved by the Stanford University Animal Care and Use Committee (IACUC). Female athymic nude mice (nu/nu) in 4–6 weeks old were obtained from the Charles River Laboratories (Boston, MA, USA) and kept under sterile conditions. MCF-7-TYR or MCF-7 cells (1 × 10^7^) suspended in 100 μL of PBS were inoculated subcutaneously in the shoulder of nude mice. When the tumors reached 0.5–0.8 cm in diameter, the tumor bearing mice were subjected to *in vivo* multimodality imaging (PAI, MRI and PET) and biodistribution studies.

### PAI and MRI of tumor bearing mice

Mice bearing different tumors (MCF-7-TYR, or MCF-7) were anesthetized with 2% isoflurane in oxygen and placed with lateral position. MRI was performed using the same instrument, protocols and conditions as in the phantom MRI study. Imaging analysis was performed using the OsiriX software. The contrast was adjusted and ROIs were drawn over the tumor and muscle. T1 value of ROIs was then measured, and the ratio of tumor/muscle was calculated. PAI was carried out using the same Vevo LAZR PAI System as the *in vitro* study. Similarly, image analysis was carried out using ImageJ, and quantification analysis was performed on the PAI images.

### Small-animal PET

Small animal PET imaging of tumor-bearing mice was performed on a micro-PET R4 rodent scanner (Siemens Medical Solutions Inc., Knoxville, TN, USA). Mice bearing MCF-7-TYR or MCF-7 tumors were injected with ^18^F-P3BZA (4.09 ± 0.07 MBq, 110.5 ± 2.0 μCi) via their tail vein. At different times after injection (0.5, 1, and 2 h), the mice were anesthetized with 2% isoflurane and placed prone near the center of the FOV of the scanner. Three-minute static scans were obtained. All the small animal PET images were reconstructed by a two-dimensional ordered-subsets expectation maximization (OSEM) algorithm. No background correction was performed.

### Biodistribution studies

For biodistribution studies, mice bearing MCF-7-TYR or MCF-7 xenografts (n = 4 for each group) were sacrificed at 2 h after tail vein injection of 0.74 MBq (20 μCi) of ^18^F-P3BZA. Tumors and normal tissues of interest were removed and weighed, and radioactivity was measured by gamma-counter. The radioactivity uptake in the tumor and normal tissues was expressed as a percentage of the injected radioactive dose per gram of tissue (%ID/g).

### Statistics

Statistical analysis was performed using the Student t test. A 95% confidence level was chosen to determine the significance of differences between groups, with a *P* value of less than 0.05 indicating a significant difference.

## Author Contributions

Z.C. conceived and designed the study devised, supervised the project, and wrote the manuscript. C.Q. designed the study, performed all the experiments, and wrote the manuscript. K.C. contributed to the design the plasmid and cell study. Y.L. and H.L. performed the radiosynthesis of the PET probe. K.C., X.H., L.B., X.S., X.Z. and S.M. contributed to animal biodistribution and imaging study. X.L., Y.Z. and Y.X. contributed to the study design and preparation of the manuscript.

## Figures and Tables

**Table 1:** Biodistribution results for ^18^F-P3BZA at 2 h post-injection in MCF-7-TYR or MCF-7 tumor bearing mice (n = 4 for each group). Data are expressed as the percentage administered activity (injected dose) per gram of tissue (%ID/g). Significant difference between MCF-7-TYR and MCF-7 tumor uptake was observed (*P* < 0.05)

Organ (%ID/g)	MCF-7-TYR	MCF-7
Tumor	11.80 ± 1.60	0.52 ± 0.06
Blood	0.16 ± 0.03	0.10 ± 0.01
Heart	0.42 ± 0.06	0.39 ± 0.06
Lungs	0.48 ± 0.10	0.53 ± 0.12
Liver	1.71 ± 0.21	2.32 ± 0.27
Spleen	0.71 ± 0.15	0.42 ± 0.12
Pancreas	0.46 ± 0.10	0.41 ± 0.09
Stomach	1.12 ± 0.20	0.49 ± 0.07
Brian	0.37 ± 0.07	0.20 ± 0.04
Intestine	0.66 ± 0.08	0.63 ± 0.24
Kidneys	1.17 ± 0.24	0.53 ± 0.24
Skin	0.48 ± 0.08	0.26 ± 0.08
Muscle	0.34 ± 0.04	0.24 ± 0.04
Bone	0.41 ± 0.06	0.25 ± 0.06
**Uptake Ratio**		
Tumor/Blood	76.28 ± 12.02	5.62 ± 1.25
Tumor/Muscle	34.41 ± 6.33	2.29 ± 0.64
